# From algorithm performance to clinical value in AI-assisted skin cancer diagnostics

**DOI:** 10.3389/fdgth.2026.1896521

**Published:** 2026-10-12

**Authors:** Lennart Ocker-Serger, Elisabeth Livingstone, Georg Lodde, Eggert Stockfleth, Nessr Abu Rached, Dirk Schadendorf, Stephan Nüesch

**Affiliations:** 1 Department of Dermatology, Allergology and Venerology, University Hospital Essen, Bochum, Germany; 2 Department of Dermatology, Allergology and Venerology, Ruhr-University Bochum, Bochum, Germany; 3 Department of Dermatology, Bielefeld University, Medical School and University Medical Center OWL, Klinikum Bielefeld Rosenhöhe, Bielefeld, Germany; 4 International Institute of Management in Technology (IIMT), University of Fribourg, Fribourg, Switzerland

**Keywords:** artificial intelligence, clinical utility, digital health, melanoma, skin cancer, value-based healthcare

## Abstract

**Background:**

Artificial intelligence (AI) has demonstrated high diagnostic performance in skin cancer detection and is increasingly being integrated into clinical workflows. However, whether these advances translate into meaningful patient-relevant and healthcare system outcomes remains unclear. Evidence on value-oriented outcomes has not been systematically synthesized.

**Methods:**

We conducted a PRISMA-ScR–guided scoping review of studies published between January 2015 and October 2025 identified through PubMed/MEDLINE, Scopus, and Web of Science. Studies evaluating AI-based technologies for skin cancer diagnosis, triage, risk stratification, or management were included if they reported outcomes beyond technical performance metrics. Reported outcomes were mapped to three value-oriented domains: clinical and prognostic, process and experiential, and system-level and economic outcomes.

**Results:**

Of 4,021 records identified, 33 studies met the inclusion criteria, whereas 389 publications were excluded because they reported only technical performance metrics without value-oriented outcomes. Reported outcomes were predominantly process and experiential (28/33 studies), including diagnostic confidence, workflow efficiency, and user acceptance. Clinical and prognostic outcomes were reported in only five studies, with four studies focussing on prognostic utility and one study focussing on patient management, while system-level and economic outcomes were identified in three studies. Most evaluations were conducted in controlled experimental settings, whereas prospective real-world implementation studies were less common. Overall, the available evidence predominantly focused on process-level benefits, with limited evidence for clinical and prognostic or system-level and economic outcomes.

**Discussion:**

Current evaluation strategies for AI-based skin cancer diagnostics remain predominantly performance-oriented and are largely focused on process and experiential outcomes. From a value-based healthcare perspective, current evidence remains largely confined to intermediate stages of the diagnostic value chain: although AI-assisted systems may improve diagnostic workflows, clinician confidence, and clinical decision making, few studies have evaluated their impact on downstream patient-relevant clinical and prognostic, or system-level and economic outcomes. Future research should prioritize prospective real-world studies, outcome-driven evaluation frameworks, and economic assessments that examine the impact of AI-supported decision-making on clinical outcomes and healthcare systems.

**Conclusion:**

Despite promising effects on diagnostic processes, evidence for the clinical and system-level value of AI-based skin cancer diagnostics remains limited. Outcome-driven evaluation frameworks are needed to bridge the gap between algorithmic performance and meaningful healthcare impact.

## 1 Introduction

Skin cancer represents one of the most prevalent malignancies worldwide, with rising incidence rates for both melanoma and non-melanoma skin cancers contributing to a substantial and growing burden on healthcare systems (1–3). Early and accurate diagnosis is a key determinant of prognosis, treatment intensity, and long-term healthcare costs (4). However, increasing case numbers, limited dermatological resources, and pronounced variability in diagnostic expertise across care settings continue to pose major challenges in routine clinical practice, particularly in primary care and teledermatological contexts (5, 6).

Over the past decade, artificial intelligence (AI), particularly deep learning-based image analysis, has emerged as a promising technological approach to support skin cancer diagnostics (7). Numerous studies have demonstrated high diagnostic performance of AI systems in the classification of pigmented skin lesions, in some cases matching or exceeding the accuracy of experienced dermatologists under controlled conditions (8–10). Consequently, AI-assisted diagnostic tools are increasingly discussed as potential solutions to improve early detection, optimize triage, and expand access to specialist care, particularly in settings with limited dermatological expertise (11, 12).

Despite this rapid technological progress, the existing literature on AI in skin cancer diagnostics is largely characterized by a strong focus on technical performance metrics (13, 14). While these metrics are essential for model development and benchmarking, their relevance for clinical practice is limited (15). High diagnostic performance observed under experimental reader-study conditions does not necessarily translate into improved clinical decision-making, better patient outcomes, or more efficient care pathways when AI systems are implemented in real-world settings (15, 16). Consequently, the actual clinical and health system impact of AI-based diagnostic technologies remains insufficiently characterized.

Previous systematic reviews have comprehensively synthesized the diagnostic performance of AI-based skin cancer detection systems, focusing on measures such as sensitivity, specificity, and algorithm accuracy (13, 14). However, they provide limited insight into whether high technical performance translates into improvements in patient care, healthcare processes, or system-level value. From a value-based healthcare (VBHC) perspective, the evaluation of diagnostic innovations extends beyond technical performance to outcomes that matter to patients and health systems, including downstream clinical consequences, diagnostic processes, patient experience, and resource utilization (17–20).

The aim of this review was to systematically map and synthesize value-oriented outcomes reported for AI-based diagnostic technologies in skin cancer care using a VBHC-informed framework. By categorizing the literature according to predefined outcome domains, this review complements existing performance-focused reviews by providing a structured overview of where, how, and under which conditions value-oriented outcomes have been evaluated in AI-based skin cancer diagnostics.

## 2 Methods

### 2.1 Study design and reporting standards

This review was conducted as a scoping review, following the methodological framework proposed by Arksey and O'Malley and reported in accordance with the PRISMA Extension for Scoping Reviews (PRISMA-ScR) (21, 22). A scoping review approach was chosen to systematically map and characterize the heterogeneous literature on artificial intelligence (AI) applications in skin cancer diagnostics with respect to value-oriented reporting, rather than to quantitatively synthesize intervention effects or estimate pooled diagnostic performance. Unlike previous systematic reviews that primarily focused on diagnostic accuracy and algorithm performance, this review aimed to examine how clinical, process-related, and system-level outcomes have been assessed following or alongside technical validation of AI systems. To support a structured synthesis, reported outcomes were interpreted using a VBHC-informed conceptual framework. Consequently, studies exclusively reporting technical performance metrics without clinical, process and experiential, or system-level and economic outcomes were excluded. No review protocol was prospectively registered or published.

### 2.2 Eligibility criteria and outcome framework

Studies were eligible for inclusion if they met the following criteria:

(1) evaluated AI-based technologies applied to the diagnosis, triage, risk stratification, or management of skin cancer;
(2) involved human participants or clinically relevant datasets;
(3) reported at least one value-oriented outcome beyond standalone technical performance metrics (4), were published in peer-reviewed journals in English or German.

Conference abstracts, editorials, comments, animal studies, and publications without original data were excluded. Narrative reviews and systematic reviews were not included in the evidence synthesis but were used to identify relevant background literature and additional primary studies through citation tracking, where appropriate. Importantly, no additional eligible studies were identified through citation tracking, and all included studies were retrieved through the described database search strategy.

Value-oriented outcomes were defined as outcomes extending beyond standalone measures of algorithmic performance and reflecting consequences for clinical decision-making, patient care, healthcare processes, or health systems. Studies reporting only technical performance metrics (e.g., accuracy, sensitivity, specificity, AUROC) were excluded. Studies reporting technical performance together with value-oriented outcomes were eligible for inclusion. To support a structured synthesis, outcomes were classified into predefined outcome domains using a VBHC-informed conceptual framework. Outcome domains were assigned according to the level at which the consequences of AI use were measured.

- Clinical and prognostic outcomes: outcomes reflecting either
  (i) patient management, directly measured downstream consequences for patient care or clinical management (e.g., changes in management, diagnostic procedures, or treatment pathways), or
  (ii) prognostic utility, including AI-based risk stratification or survival prediction. Prognostic outcomes were considered clinically relevant because they may inform clinical decision-making but were distinguished from directly demonstrated patient benefit.
- Process and experiential outcomes: intended or simulated changes in clinician decision-making, diagnostic confidence, workflow efficiency, user acceptance, trust, or patient experience.
- System-level and economic outcomes: outcomes related to healthcare utilization, resource use, costs, access to care, or broader organizational effects.

Studies reporting outcomes across multiple domains were assigned to each applicable category. Consequently, outcome-domain counts are not mutually exclusive.

### 2.3 Literature search and study selection

A comprehensive literature search was performed across PubMed/MEDLINE, Scopus and Web of Science, covering publications from January 1, 2015 to October 31, 2025. The databases were searched on November 10, 2025. The search period was restricted to publications from January 2015 onward to capture the emergence of contemporary deep learning–based AI applications in dermatology.

The outcome-related search block intentionally employed broad outcome-related terminology rather than specifying individual downstream outcome measures, which were subsequently identified during study screening and classified according to the predefined outcome framework. The search strategy combined controlled vocabulary terms (where applicable) and free-text keywords related to *artificial intelligence*, *machine learning*, *deep learning*, *skin cancer*, *melanoma*, *non-melanoma skin cancer*, *diagnosis*, and *value-oriented outcomes*. Search strategies were adapted to the syntax of each database while maintaining equivalent conceptual search domains. The complete search strategies for all databases are provided in Supplementary Table S1.

All references were imported into Rayyan (Qatar Computing Research Institute, Doha, Qatar) for deduplication and study screening. Title and abstract screening, full-text assessment, and data extraction were performed by the first author using predefined eligibility criteria. Methodological supervision was provided throughout the review process by the senior author, who reviewed the overall review methodology and resolved uncertainties regarding study eligibility and outcome classification through discussion until consensus was reached.

### 2.4 Data extraction and synthesis

For each included study, data were extracted using a standardized extraction form. Extracted variables included publication characteristics, study design, population and sample size, clinical setting, imaging modality, AI application and model, comparator (if applicable), reported value-oriented outcomes, and key study findings. All included studies were independently re-evaluated and data were extracted by two reviewers, including study characteristics, evaluation setting, primary endpoints, outcome-evaluation type, and outcome-domain assignment. Any discrepancies were resolved by consensus, with consultation of the senior author where necessary.

Given the heterogeneity of study designs, interventions, and reported endpoints, a quantitative synthesis was not considered appropriate. Instead, a narrative synthesis was conducted. Findings were synthesized according to outcome domain, study design, evaluation setting (experimental versus real-world), AI application, and reported direction of effect to identify recurring patterns and evidence gaps.

The VBHC-informed framework was used to organize reported outcomes across different dimensions of healthcare value rather than to perform a formal value-based economic evaluation. Process and experiential outcomes were interpreted as intermediate mechanisms that may contribute to value but do not in themselves demonstrate improvements in patient outcomes or resource utilization.

## 3 Results

### 3.1 Study selection and characteristics

The systematic literature search yielded 4021 records. After removal of 1,027 duplicates, 2,994 records underwent title and abstract screening. Following exclusion of 2,382 records, 612 full-text reports were sought for retrieval, of which 503 were assessed for eligibility. Thirty-four reports representing 33 studies met the predefined inclusion criteria (Figure 1).

**Figure 1:**
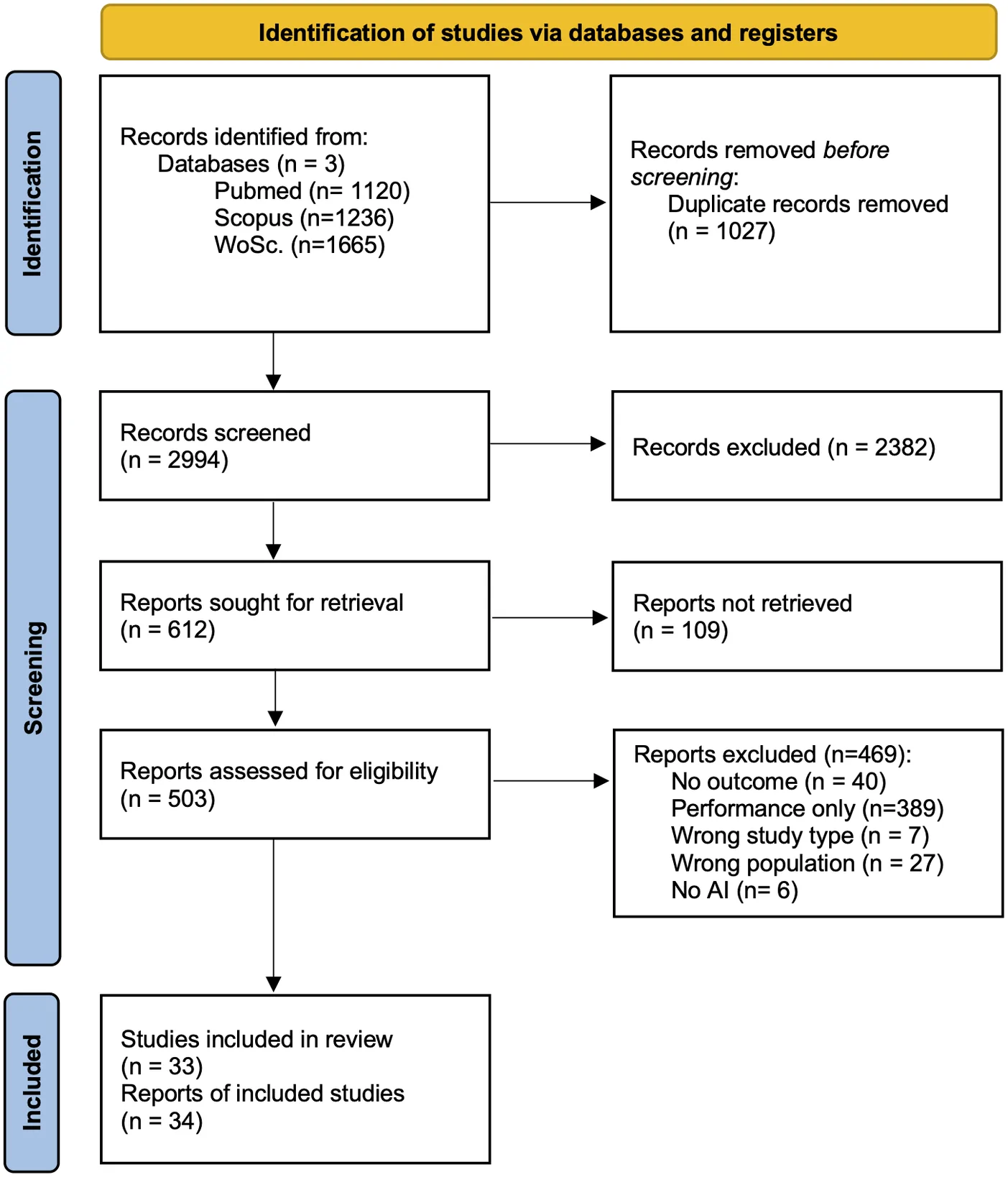
PRISMA 2020 flow diagram of study selection. Flow diagram illustrating the identification, screening, eligibility assessment, and inclusion of studies evaluating artificial intelligence applications in skin cancer diagnostics. Following database searches and duplicate removal, records were screened at title and abstract level. Full-text reports were assessed against predefined eligibility criteria, including the requirement to report at least one clinical and prognostic, process and experiential, or system-level and economic outcome beyond technical performance metrics. Publications exclusively reporting diagnostic performance measures without value-oriented outcomes were excluded. In total, 33 studies (34 reports) were included in the final synthesis. Adapted from the PRISMA 2020 flow diagram (Page et al., 2021), licensed under CC BY 4.0. Source: PRISMA Statement, PRISMA 2020 flow diagram.

The included studies encompassed a heterogeneous range of study designs, predominantly clinical studies and reader studies with fewer randomized controlled trials and health economic modelling studies. A substantial proportion of studies evaluated AI systems under controlled reader-study or experimental conditions (*n* = 20), whereas fewer investigations were embedded in real-world clinical care settings (*n* = 13).

With regard to modalities of AI input, the majority of studies focused on clinical photographs (*n* = 15) and dermoscopic images (*n* = 15), followed by applications in pathology (*n* = 3) and teledermatology (*n* = 3). Applications involving multimodal imaging or non-standard acquisition techniques were comparatively infrequent. A detailed overview of the study selection process is provided in the PRISMA flow diagram (Figure 1), and key characteristics of the included studies are summarized in Supplementary Table S2.

### 3.2 Distribution of reported outcomes

Across the included studies, reported outcomes were distributed unevenly across the predefined outcome domains. Process and experiential outcomes constituted the most frequently assessed outcome category, whereas clinical and prognostic outcomes, and system-level and economic outcomes were reported substantially less often.

Only a limited number of studies evaluated clinical or prognostic outcomes (*n* = 5), including changes in management or prognostic stratification. These studies predominantly originated from real-world clinical implementations or dermatopathological prognostic modelling. The majority of the included literature focused on workflow-related effects (*n* = 28), including diagnostic decision support, triage processes, user acceptance, trust, and perceived diagnostic confidence among clinicians and patients, often assessed in reader studies or controlled experimental settings. System-level and economic outcomes, including resource utilization, cost implications, or broader efficiency effects, were reported in three studies and were predominantly derived from economic modelling or observational analyses, rather than prospectively collected cost data.

### 3.3 Clinical and prognostic outcomes

Clinical and prognostic outcomes were reported in a limited subset of the included studies (*n* = 5). Four studies evaluated prognostic utility through AI-based risk stratification, whereas one study reported directly observed downstream effects on patient-management.

Four studies addressed prognostic outcomes, including the prediction of metastatic risk or survival probabilities. AI-derived features improved risk stratification compared with conventional approaches and provided additive prognostic information, for example by differentiating high- and low-risk patient groups relevant for clinical decision-making (23–26). For example, AI-based quantification of tumor-infiltrating lymphocytes (TIL) was associated with improved prognostic discrimination and prediction of survival (HR 0.45, 95% CI 0.26–0.80; *p* = 0.005) (23). Similarly, a deep learning model based on routine whole-slide images emerged as an independent prognostic factor (HR 1.84, *p* < 0.005) and stratified patients into low- and high-risk groups with distinct 5-year overall survival (24). In another prospective cohort study, a multimodal CNN predicted the risk of metastatic progression and disease-free survival in patients with melanoma using dermoscopy images, clinical data, serological, and histopathological biomarkers (26).

Observed clinical outcomes with effects on patient management were reported in only one prospective clinical study. AI-assisted decision-making reduced unnecessary excisions of benign nevi by 19.2% (from 104/190 to 84/190 lesions; *p* < 0.001) while maintaining complete melanoma detection, demonstrating an improvement in patient management without compromising diagnostic safety (27).

### 3.4 Process and experiential outcomes

Outcomes related to diagnostic process quality and user experience were reported substantially more frequently than clinical and prognostic outcome measures and represented the predominant focus of the included studies (*n* = 28). These outcomes primarily addressed changes in diagnostic workflows, decision-making processes, and the perception, acceptance, and use of AI-based systems by clinicians and patients.

The majority of studies in this outcome category evaluated the impact of AI-supported decision assistance on diagnostic confidence, decision quality, and interobserver consistency. Across multiple reader and clinical studies, AI-assistance, predominantly implemented as human-with-AI or augmented intelligence approaches, was associated with increased diagnostic confidence and more consistent assessments, particularly among less experienced clinicians (27–31). In a randomized controlled trial, AI assistance significantly improved the diagnostic accuracy of nonexpert physicians compared with unaided assessment (53.9% vs. 43.8%; *p* = 0.019), with the greatest benefit observed among non-dermatology trainees (54.7% vs. 30.7%; *p* < 0.0001). AI assistance also increased the number of differential diagnoses considered (2.09 vs. 1.95; *p* = 0.0005) (29). In a separate reader study, explainable AI did not further improve diagnostic accuracy compared with conventional AI support, but increased clinicians' diagnostic confidence by 12.3% and trust in the system by 17.5% (both *p* < 0.001) (32). In a further prospective clinical study, physician diagnostic accuracy increased from 74.1% to 86.4% with AI assistance (*p* < 0.001), with the greatest improvement observed among less experienced dermatologists (27).

Improvements in diagnostic workflows were reported in three studies, including more efficient triage, time savings, and potential relief of specialist services through the identification of low-risk cases, particularly in teledermatology and primary care settings (33–35). In a prospective real-world implementation study, the AI-supported pathway identified 15%–31% of assessed lesions as eligible for discharge, thereby reducing specialist workload while maintaining clinical safety (33). In one prospective teledermatology trial, an AI-based medical device identified significantly more lesions that did not require biopsy or urgent face-to-face dermatologist review than standard teledermatology (*p* = 0.001), while maintaining comparable sensitivity for skin cancer (34).

Experiential outcomes were reported in ten studies and consistently highlighted stakeholder preferences regarding AI integration. Both clinicians and patients favoured assistive AI systems over autonomous decision-making, with AI predominantly perceived as a supportive tool rather than a replacement for clinical expertise (36, 37). In one prospective cohort study, 83.4% of patients preferred augmented intelligence–based skin cancer screening over AI or dermatologist assessment alone, and AI-assisted examination was associated with a higher perceived sense of safety (38). Despite high confidence in AI-generated results, dermatologists continued to rely primarily on their own clinical judgment when assessments diverged (38). Reported barriers to adoption included concerns about diagnostic reliability, transparency, data protection, and responsibility in cases of discordant human–AI assessments (39, 40).

### 3.5 System-level and economic outcomes

System-level and economic outcomes were reported in a minority of the included studies (*n* = 3). Reported outcomes addressed potential effects on healthcare systems, resource utilization, and cost structures associated with the implementation of AI-based skin cancer diagnostics. Overall, these outcomes were predominantly evaluated using modelling approaches or conceptual analyses, with only limited empirical validation in real-world clinical settings. Potential economic implications were addressed in three studies (33, 34, 41). While one study performed a formal cost-effectiveness analysis using an economic model, demonstrating only marginal cost savings for AI-supported melanoma detection with comparable health outcomes (41), the remaining studies discussed possible cost savings indirectly through reductions in referrals, specialist consultations, or workload (33, 34).

Conversely, one study highlighted potential negative system-level implications, including overdetection, unnecessary procedures or referrals, and increased healthcare utilization resulting from inaccurate or insufficiently validated AI systems. However, these effects were discussed qualitatively but were not quantified using formal economic or system-level analyses (40).

### 3.6 Validation approaches and comparison strategies

Validation strategies varied substantially across studies. Most studies relied on internal validation procedures, such as train–test splits or cross-validation, or reader-based comparison designs, whereas prospective external validation in real-world clinical settings was uncommon. Human-with-AI comparisons represented the most frequently applied evaluation strategy, while comparisons against routine clinical pathways were rare.

## 4 Discussion

### 4.1 Clinical value of AI-based skin cancer diagnosis

Across the reviewed literature, artificial intelligence is frequently positioned as a transformative innovation for skin cancer diagnostics, primarily on the basis of high diagnostic performance in controlled evaluation settings (13, 42). However, the findings of this review reveal a systematic disconnect between reported model performance and empirically demonstrated clinical value. While diagnostic accuracy metrics are widely reported, evidence beyond technical performance predominantly focuses on process and experiential outcomes, whereas prognostic utility and directly observed patient management outcomes remain scarce. Four studies focused on prognostic utility through AI-based risk stratification, whereas only one prospective study demonstrated directly observed downstream effects on patient management.

From a VBHC perspective, value in diagnostics is not an intrinsic property of an algorithm but emerges only through its influence on downstream decisions and care pathways (18, 43). Clinical value arises when diagnostic outputs improve downstream decision-making and ultimately contribute to earlier detection, avoidance of unnecessary interventions, improved patient experience, efficient care processes or resource utilization (16, 44). Improved risk stratification represents clinically relevant prognostic information. However, its impact on patient outcomes depends on whether these predictions translate into changes in clinical management and downstream care.

A differentiated analysis across outcome domains revealed pronounced imbalances in the current evidence base. Clinical or prognostic outcomes were reported only rarely (23–27), whereas process and experiential outcomes predominated across reader- and experimental studies (28, 45). System-level and economic outcomes remained scarce and were largely based on modelling approaches rather than prospective evaluations (33, 34, 41). The predominance of process and experiential outcomes and the relative scarcity of robust clinical and prognostic, or system-level and economic outcomes suggest that current evaluations of AI-based skin cancer diagnostics remain concentrated at intermediate stages of the diagnostic value chain (Figure 2). Consequently, reliance on performance benchmarks and surrogate workflow indicators may overestimate clinical value and obscure the conditions under which AI can meaningfully contribute to improved patient care. This interpretation is further supported by several studies reporting neutral or mixed findings, including no improvement in diagnostic accuracy despite increased clinician confidence, only marginal economic benefits, and potential unintended consequences such as unnecessary referrals or overdiagnosis (32, 40, 41).

**Figure 2:**
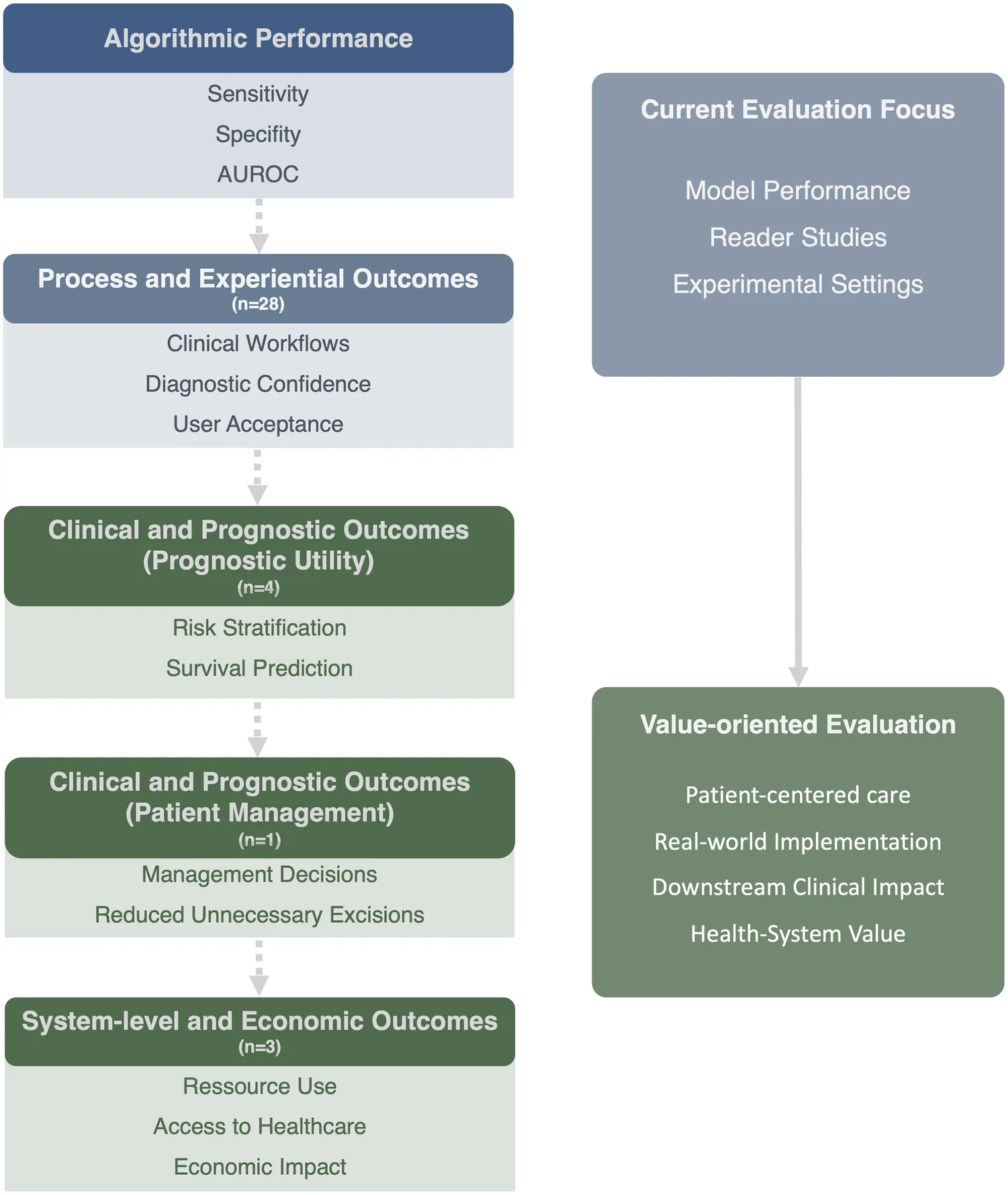
Translational gap between algorithmic performance and value-oriented clinical evaluation in AI-based skin cancer diagnostics. Conceptual model illustrating the progression from algorithmic performance to clinical and prognostic, and system-level and economic outcomes. Most studies focus on technical discrimination metrics, whereas substantially fewer evaluate process and experiential, clinical and prognostic (patient management or prognostic utility), or system-level and economic outcomes. Numbers indicate the distribution of reported outcomes among included studies. The right panel contrasts the prevailing performance-focused evaluation paradigm with a value-oriented approach emphasizing patient-centered outcomes and real-world implementation. The dotted arrows illustrate a conceptual pathway from algorithmic performance to value-oriented evaluation and do not represent an empirically demonstrated progression.

### 4.2 Interpreting process and experiential outcomes

The predominance of process and experiential outcomes in the current evidence base requires careful clinical interpretation. Across multiple studies, AI-assisted diagnostic systems were associated with increased diagnostic confidence, improved interobserver consistency, enhanced workflow efficiency, and greater user acceptance, particularly in human-with-AI configurations (27–29, 31, 34). These findings indicate that AI technologies may have the potential to influence diagnostic processes and professional experience. However, only one study demonstrated that such process-level effects translated into measurable improvements in clinically consequential endpoints, represented by a reduction of unnecessary biopsies or excisions (27).

Performance metrics primarily reflect the technical discrimination capacity of a model within a defined dataset and do not capture whether or how AI-generated predictions influence clinical decision-making in practice (46, 47). Evaluating clinical utility therefore requires a causal evaluation framework that explicitly examines how AI systems are embedded into decision processes and what downstream consequences arise for diagnostic, therapeutic, and patient-relevant outcomes (16). Importantly, the clinical impact of AI applications is highly context-dependent and shaped by deployment settings, user expertise, existing workflows, and organizational structures (44). Diagnostic performance observed in controlled experimental or reader-studies cannot therefore be directly extrapolated to real-world care pathways, where human judgment, risk tolerance, and resource constraints influence decision-making (44, 48). Evidence from adjacent technological domains illustrates that process modification does not necessarily yield improved patient outcomes. Large-scale implementation of total-body photography in high-risk populations has been associated with increased diagnostic activity and biopsy rates without proportional improvements in melanoma detection, illustrating how technological expansion may intensify intervention without clear patient-level benefit (49, 50).

Bridging the gap between model performance and clinical impact therefore requires evaluations that examine how algorithmic predictions influence clinical decisions and downstream patient and system-level outcomes under real-world conditions (15). Accordingly, future studies should evaluate AI tools under real-world conditions and explicitly measure patient-centered and system-level endpoints rather than relying solely on performance metrics. The predominance of process and experiential outcomes in the literature should therefore be interpreted as evidence of an early stage of clinical evaluation rather than as evidence that AI lacks clinical value (15, 16, 51).

### 4.3 Implementation, acceptance, and trust

The translation of AI-based diagnostic systems from technical feasibility to clinical value depends not only on performance characteristics but critically on implementation conditions, user acceptance, and trust. Across multiple studies, both clinicians and patients expressed a clear preference for augmented intelligence models in which AI supports rather than replaces human decision-making (36–38). Human oversight is consistently regarded as essential for contextual interpretation, accountability, and responsibility in cases of diagnostic discordance (38).

Acceptance remains ambivalent. While AI assistance has been associated with increased diagnostic confidence and perceived workflow efficiency, concerns regarding reliability, data protection, and regulatory responsibility persist (36, 37). The phenomenon of algorithm aversion illustrates that users may disproportionately penalize algorithmic errors compared to human mistakes, even when objective performance is superior (52). In this context, explainable AI approaches have been proposed to enhance transparency and mitigate distrust by making algorithmic outputs more interpretable (32, 53). While explainability may strengthen confidence and facilitate integration into diagnostic workflows, it does not in itself establish improved patient outcomes.

Implementation context further shapes impact. Clinical setting, user expertise, workflow integration, and regulatory frameworks determine how AI predictions are operationalized and whether they translate into measurable benefits (44). AI systems therefore function as components of sociotechnical systems, in which value arises only when algorithmic outputs are meaningfully connected to patient-centered decisions and downstream outcomes.

The available evidence should be interpreted with caution. Most included studies were retrospective reader-based, experimental, or modelling studies, frequently conducted within single centers or specific healthcare systems, whereas prospective multicenter implementation studies and randomized clinical trials were scarce. Consequently, both the generalizability and the strength of the available evidence remain limited, restricting robust conclusions regarding the effectiveness of AI-based skin cancer diagnostics in routine clinical practice.

This review has several limitations. As a scoping review, its primary objective was to map and characterize the existing literature rather than to quantitatively synthesize evidence or determine the effectiveness of AI-based technologies. Accordingly, no formal risk-of-bias assessment or meta-analysis was performed, and the findings should be interpreted as an overview of outcome reporting rather than as an assessment of the certainty or quality of evidence. Furthermore, title and abstract screening and full-text eligibility assessment were initially performed by a single reviewer under methodological supervision of the senior author, which may have increased the risk of study-selection bias. However, all included studies were subsequently independently re-evaluated and study-level data were extracted by two reviewers, with discrepancies resolved by consensus and consultation of the senior author where necessary. The mapping of outcomes to predefined domains represents a structured but interpretative classification, which may be influenced by reporting variability within primary studies. In addition, the restriction to peer-reviewed publications in selected databases may have excluded relevant gray literature or emerging evidence. While several studies assessed clinician and patient perspectives, broader stakeholder perspectives were only sparsely represented in the available literature. Nevertheless, the strength of this review lies in its systematic outcome-oriented synthesis and its explicit application of a value-based analytical framework, providing a structured perspective on the translational gap between technical performance and clinical impact.

### 4.4 Implications for future research and evaluation

The findings of this review underscore the need for a shift in how AI-based diagnostic systems are evaluated. Future research should move beyond performance benchmarking and explicitly adopt outcome-driven evaluation frameworks that link algorithmic predictions to clinically meaningful consequences.

First, more prospective studies embedding AI tools into routine clinical workflows are required to assess how AI integration affects downstream diagnostic and therapeutic decisions. Reader studies and retrospective validation analyses provide important insights into technical feasibility, but are insufficient to establish clinical utility (54). Evaluations conducted under real-world conditions should examine effects on management decisions, referral patterns, biopsy rates, or time to treatment across different deployment contexts such as primary care, teledermatology and specialist settings.

Second, evaluation strategies should adopt explicit causal models of impact to distinguish intermediate workflow effects from true improvements of clinical and prognostic, or system-level and economic outcomes. Rather than assuming that improved discrimination performance implies improved care, future investigations should define in advance which clinical decisions are expected to change, how those changes are operationalized, and which outcomes will be affected (16).

Third, system-level and economic endpoints should be systematically incorporated into future research. Formal cost-effectiveness analyses, measurement of healthcare utilization, and long-term follow-up of patient trajectories are necessary to determine whether AI-based diagnostics generate sustainable value within constrained health systems. Without such evidence, claims of transformative impact remain speculative.

AI-based skin cancer diagnostics hold considerable promise. Realizing this potential, however, requires evaluation paradigms that align technological innovation with patient-centered outcomes, contextual implementation, and accountable resource use.

## Data Availability

No primary datasets were generated for this study. Extracted study characteristics and value-oriented outcomes are summarized in Supplementary Table S2.
