# Supplementary material for: From algorithm performance to clinical value in AI-assisted skin cancer diagnostics
Source: Front Digit Health. 2026 Oct 12;8:1896521. doi: 10.3389/fdgth.2026.1896521 (PMC13696442; doi:10.3389/fdgth.2026.1896521)
Supplement: Supplementary file 1 [file Table1.pdf]

1 Supplementary Material

Database	Search date	Publication period	Records retrieved
PubMed/MEDLINE	10 Nov 2025	1 Jan 2015 – 31 Oct 2025	1120
Scopus	10 Nov 2025	2015 – 2025	1236
Web of Science Core Collection	10 Nov 2025	2015 – 2025	1665

Database specific search strategies

Database	Search strategy	Applied Filters
PubMed	("artificial intelligence"[Title/Abstract] OR AI[Title/Abstract] OR "machine learning"[Title/Abstract] OR "deep learning"[Title/Abstract] OR neural network*[Title/Abstract] OR convolutional neural network*[Title/Abstract] OR "CNN"[Title/Abstract] OR DNN[Title/Abstract]) AND ("skin cancer"[Title/Abstract] OR melanoma[Title/Abstract] OR "cutaneous melanoma"[Title/Abstract] OR "basal cell carcinoma"[Title/Abstract] OR BCC[Title/Abstract] OR "squamous cell carcinoma"[Title/Abstract] OR SCC[Title/Abstract] OR "cutaneous squamous cell carcinoma"[Title/Abstract] OR "cSCC"[Title/Abstract]) AND ("patient outcome*"[Title/Abstract] OR outcome*[Title/Abstract] OR "patient-reported outcome*"[Title/Abstract] OR "quality of life"[Title/Abstract] OR "diagnostic	Publication date: 1 January 2015 – 31 October 2025; Language: English or German;<br>Species: Humans;<br>Document type: Article, Review

	accuracy"[Title/Abstract] OR "value-based healthcare"[Title/Abstract] OR "VBHC"[Title/Abstract]) AND ("2015/01/01"[Date - Publication] : "2025/10/31"[Date - Publication]) AND (english[Language] OR german[Language]) AND (humans[MeSH Terms])	
Scopus	TITLE-ABS-KEY ("artificial intelligence" OR AI OR "machine learning" OR "deep learning" OR "neural network*" OR "convolutional neural network*" OR "CNN" OR DNN) AND TITLE-ABS-KEY ("skin cancer" OR melanoma OR "cutaneous melanoma" OR "basal cell carcinoma" OR BCC OR "squamous cell carcinoma" OR SCC OR "cutaneous squamous cell carcinoma" OR cSCC) AND TITLE-ABS-KEY ("patient outcome*" OR outcome* OR "patient-reported outcome*" OR "quality of life" OR "diagnostic accuracy" OR "value-based healthcare" OR VBHC) AND PUBYEAR > 2014 AND PUBYEAR < 2026	Publication years: 2015–2025;<br>Language: English or German;<br>Document type: Article, Review
Web of Science	TS=(("artificial intelligence" OR AI OR "machine learning" OR "deep learning" OR neural network* OR "convolutional neural network*" OR CNN OR DNN)<br>AND<br>("skin cancer" OR melanoma OR "cutaneous melanoma" OR "basal cell carcinoma" OR BCC OR "squamous cell carcinoma" OR SCC OR "cutaneous squamous cell carcinoma" OR cSCC)<br>AND<br>("patient outcome*" OR outcome* OR "patient-reported outcome*" OR "quality of life" OR "diagnostic accuracy" OR "value-based healthcare" OR VBHC))	Publication years: 2015–2025;<br>Language: English or German;<br>Document type: Article, Review, Proceedings Paper
