# Supplementary material for: From algorithm performance to clinical value in AI-assisted skin cancer diagnostics
Source: Front Digit Health. 2026 Oct 12;8:1896521. doi: 10.3389/fdgth.2026.1896521 (PMC13696442; doi:10.3389/fdgth.2026.1896521)
Supplement: Supplementary file 2 [file Table2.docx]

| **Author, Year** | **Study Design** | **Setting** | **Population / N** | **AI input** | **AI task** | **Comparator** | **Primary Endpoint(s)** | **Main Outcome domain(s)** | **Outcomes Evaluation** | **Key value-related findings** |
| --- | --- | --- | --- | --- | --- | --- | --- | --- | --- | --- |
| Bramer et al., 2025  (1) | Retrospective nested case control study | Experimental | 374 patients with cSCC (187 cases and 187 controls; 130 development, 244 evaluation cohort) | Pathology whole-slide images | Feature Extraction  and risk stratification | Conventional histopathology | Prediction of metastatic risk | Clinical (prognostic utility) | Model-based | Improved prognostic stratification and identification of high-risk cSCC patients using AI-derived histopathological features |
| Aung et al., 2025 (2) | Retrospective clinical study | Experimental | 111 patients with melanoma (prognostic cohort); 98 participants | Pathology whole-slide images | AI-based TIL-Scoring and prognostic assessment | Conventional histopathology | Reproducibility and prognostic validity of AI-assisted TIL-scoring | Clinical (prognostic utility) | Model-based | AI-based TIL scoring demonstrated superior reproducibility and prognostic prediction compared to conventional scoring |
| Jones et al., 2025 (3) | Qualitative semi-structured interview study | Experimental | 29 stakeholders (patients, primary care, dermatologists, AI researchers) | Not applicable | Not applicable | Not applicable | Perceived barriers and facilitators to AI implementation in skin cancer diagnosis | Process and experiential | Survey-based | Stakeholders identified potential benefits of AI for early skin cancer detection but emphasized trust, data quality, and workflow integration as key implementation requirements. |
| Bossard et al., 2025 (4) | Retrospective modelling study | Experimental | 566 patients with stage I–III melanoma (342 discovery, 161 external validation, 63 TCGA validation) | Pathology whole-slide images | Feature extraction  and risk stratification | Established risk models | Prediction of overall survival and prognostic risk stratification | Clinical (prognostic utility) | Model-based | AI-based histopathology evaluation improved 5-year survival prediction and prognostic risk stratification in stage I–III melanoma |
| Gaube et al., 2024 (5) | Experimental conjoint survey study | Experimental | 1,899 participants (1,591 main study; 308 replication study) | Not applicable | Not applicable | Dermatologist assessment | Preferences for AI-enabled skin cancer screening versus dermatologist screening | Process and experiential | Survey-based | Participants preferred dermatologist over AI-based screening, with greater perceived trust and quality for dermatologists. |
| Goessinger et al., 2024 (6) | Prospective clinical study | Clinical | 205 high-risk patients undergoing skin cancer screening and 8 dermatologists | Clinical photography  + Dermoscopy  + Total body photography | Melanoma risk classification | Human vs.  Human +AI | Acceptance and perceptions of augmented AI-assisted skin cancer screening | Process and experiential | Survey-based | Patients preferred augmented AI over AI or dermatologist-alone screening with higher perceived safety; dermatologists expressed high trust in AI but continued to rely primarily on their own clinical judgment. |
| Chanda et al., 2024 (7) | Prospective reader study | Experimental | 116 dermatologists | Dermoscopy | Melanoma vs. Nevus classification with explainable decision support | Human vs.  Human + AI vs.  Human + XAI | Effect of AI and explainable AI on dermatologists' diagnostic accuracy, confidence, and trust in the XAI-support | Process and experiential | Directly observed | AI support improved clinicians’ diagnostic accuracy, while XAI further increased diagnostic confidence and trust without significantly improving accuracy beyond AI alone. |
| Chanda et al., 2025(8) | Prospective reader study | Experimental | 76 dermatologists | Dermoscopy | Melanoma vs. Nevus classification with explainable decision support | Human vs.  Human + AI vs.  Human + XAI | Impat of AI and explainable AI on dermatologists´ diagnostic accuracy, interaction with AI / XAI system (ocular fixations) | Process and experiential | Directly observed | Dermatologists’ diagnostic accuracy increases with XAI over AI alone; Disagreements with the classifier decisions and complex lesions (higher difficulty) correlate with ocular fixation. |
| Winkler et al., 2023 (9) | Prospective clinical study | Clinical | 22 dermatologists; 188 patients with 228 suspect melanocytic lesions | Dermoscopy | Melanoma vs. Nevus Classification | Human vs.  Human + AI | Diagnostic performance of dermatologists with versus without CNN assistance | Clinical (patient management) + Process and experiential | Directly observed | AI assistance reduced unnecessary excisions by 19.2 % while maintaining complete melanoma detection and improved diagnostic performance, particularly among less experienced dermatologists. |
| Escalé-Besa et al., 2023 (10) | Prospective clinical study | Clinical | 100 primary care patients with skin lesions | Clinical photography  ~~+ Dermoscopy~~ | Multi-class classification of skin diseases | Standard care | Diagnostic accuracy of an ML model compared with GPs and teledermatology dermatologists | Process and experiential | Directly observed | ML diagnostic accuracy was lower than clinician performance, but was perceived as a useful diagnostic support tool by most GPs. |
| Sangers et al., 2023 (11) | Survey study | Experimental | 16 dermatologists and 17 general practitioners | Not applicable | Not applicable | Not applicable | Healthcare professionals' views on barriers, benefits, and conditions for AI implementation | Process and experiential | Survey-based | Healthcare professionals perceived potential benefits of AI in skin cancer care, but identified concerns regarding accuracy, health inequalities, and replacement, as well as requirements for usability, accessibility, validation, and regulation. |
| Gomez Rossi et al., 2022 (12) | Retrospective modelling study | Experimental | Simulated US cohort (melanoma subanalysis) | Clinical photography  + Dermoscopy | Melanoma detection | Standard care | Lifetime costs and QALYs of AI-assisted melanoma detection compared with standard care. | System-level  and economic | Model-based | AI-assisted melanoma detection was associated with marginal model-estimated cost savings and similar QALYs compared with standard care. |
| Shoham et al., 2022  (13) | Retrospective clinical study | Experimental | 8644 Mohs micrographic surgery (MMS) procedures | Structured preoperative clinical data | Surgical complexity prediction | Standard care | Prediction of MMS procedural complexity | Process and experiential | Model-based | AI predicted Mohs surgery complexity, including the number of surgical stages and wound reconstruction complexity. |
| Lim et al., 2022 (14) | Cross-sectional survey study | Clinical | 603 patients in outpatient skin cancer clinics | Not applicable | Not applicable | Not applicable | Patient perceptions toward AI-assisted skin cancer screening | Process and experiential | Survey-based | Patients preferred AI as decision support rather than replacement of clinicians |
| Nelson et al., 2020  (15) | Qualitative semi-structured interview study | Clinical | 48 dermatology patients | Not applicable | Not applicable | Not applicable | Patient perceptions toward AI-assisted skin cancer screening | Process and experiential | Survey-based | Most patients supported AI-assisted skin cancer screening, while 94% emphasized importance of human–AI symbiosis and preservation of physician involvement. |
| Tong & Sopory, 2019 (16) | Randomized expierimental messaging study | Experimental | 273 participants | Not applicable | Not applicable | Not applicable | Effect of risk and benefit messaging on intentions to use AI for skin cancer screening | Process and experiential | Survey-based | Risk communication influenced affective responses and perceptions of AI, which in turn affected intentions to use AI-based skin cancer screening.. |
| Mei et al., 2025 (17) | Prospective diagnostic reader study | Experimental | 707 patients (validation cohort); 5,572 images (training/test cohort) | Clinical photography  + Dermoscopy | Binary classification between BCC and seborrheic keratosis | Human vs.  Human + AI | Diagnostic performance of dermatologists with vs. without AI assistance for differentiating BCC from seborrheic keratosis | Process and experiential | Directly observed | AI assistance improved dermatologists' diagnostic accuracy for differentiating BCC from seborrheic keratosis. |
| Diaz-Ramón et al., 2023 (18) | Prospective cohort study | Clinical | 196 melanoma patients | Dermoscopy + Clinical data | Melanoma Classification + Prediction of metastasic risk and disease-free survival | Standard care | AI-based melanoma diagnosis and prediction of melanoma metastasis and disease-free survival | Clinical (prognostic utility) | Model-based | Multimodal AI predicted metastatic progression and disease-free survival in melanoma using clinical, serological, and histopathological biomarker |
| Thomas et al., 2023 (19) | Prospective clinical study | Clinical | 8,571 skin lesions | Clinical photography  + Dermoscopy | Skin lesion classification and triage / referral decision support | Standard care | Real-world diagnostic performance and impact on referral/discharge pathway | Process and experiential  +System-level  and economic | Directly observed | Real-world AI-supported triage identified 15-31 % of lesions as eligible for discharge, reducing specialist workload, while maintaining high sensitivity for skin cancer detection. |
| Gregoor et al., 2023 (20) | Prospective clinical study | Clinical | 50 primary care patients with skin lesions | Clinical photography | Risk stratification | Standard care | Feasibility of AI implementation in primary care, assessed by patient and GP inclusion and participation rates. | Process and experiential | Directly observed | AI-supported triage influenced GP management decisions in primary care setting and showed potential to reduce unnecessary consultations |
| Marchetti et al., 2023 (21) | Prospective reader study | Experimental | 435 patients (603 suspicious skin lesions) | Dermoscopy | Classification of melanocytic skin lesions | Human vs.  Human + AI | Diagnostic accuracy of the ADAE AI algorithm for melanoma detection | Process and experiential | Directly observed | Augmented AI improved dermatologists' melanoma risk assessment and influenced intended biopsy recommendations |
| Vollmer et al., 2025 (22) | Cross-sectional reader study | Experimental | 200 histologically confirmed rare skin tumors; international panel of dermatologists | Clinical photography  + Dermoscopy | Classification | Standard care | Diagnostic performance of a DL-CNN for rare skin tumors compared with dermatologists | Process and experiential | Directly observed | AI provided limited support in diagnosing rare skin tumors and did not improve overall diagnostic accuracy. |
| Metta et al., 2024 (23) | Survey-based user study | Experimental | 156 participants | Clinical photography | Multi-class skin lesion classification with explainable AI support | Explainable AI vs. Conventional AI | Evaluation of explainable AI for skin lesion classification and effect on trust and confidence | Process and experiential | Survey-based | Explainable AI improved trust and confidence in AI-assisted skin lesion classification. |
| Marsden et al., 2024 (24) | Prospective clinical study | Clinical | 622 patients (789 skin lesions) | Clinical photography  + Dermoscopy  + Teledermatology | Classification + Triage + Decision support | Standard care | Classification of lesions not requiring biopsy or urgent dermatology review | Process and experiential  + System-level and economic | Directly observed +  Model-based | Real-world AI triage identified more lesions not requiring biopsy/urgent referral than teledermatology at comparable sensitivity, with modeled reductions in resource use and costs |
| Felmingham et al., 2023 (25) | Prospective clinical study | Clinical | 214 participants (743 skin lesions) | Clinical photography  + Teledermatology | Classification + Decision support | Standard care | Diagnostic accuracy of CNN versus teledermatologist assessment | Process and experiential | Directly observed | AI assistance improved resident management decisions but also increased unnecessary biopsies in some benign lesions |
| Han et al., 2022 (26) | Prospective randomized controlled trial | Clinical | 576 patients with suspicious skin lesions | Clinical photography | Multi-class Classification | Human vs.  Human + AI | Diagnostic accuracy of AI-assisted versus unaided physicians | Process and experiential | Directly observed | AI assistance improved diagnostic accuracy; particularly among less experienced clinicians |
| Jahn et al., 2022 (27) | Prospective clinical study | Clinical | 114 patients (1204 pigmented skin lesions) | Clinical photography  + Teledermatology | Classification | Standard care | Diagnostic accuracy of smartphone AI app for melanoma risk stratification | Process and experiential | Directly observed | AI smartphone app showed limited real-world diagnostic performance with substantial over-detection of melanoma-suspect lesions and low user trust. |
| Ba et al.,  2022 (28) | Prospective reader study | Experimental | 25,773 clinical images (Training dataset); 2107 clinical images (validation); 400 test images (reader study); 18 dermatologists | Clinical photography | Multi-class classification | Human vs.  Human + AI | Diagnostic accuracy of CNN-assisted versus unassisted dermatologists | Process and experiential | Directly observed | AI support improved dermatologists' diagnostic accuracy, greatest benefit among less experienced dermatologists |
| Han et al., 2020 (29) | Retrospective reader study | Experimental | 220,680 images (training dataset); main reader test: 240 images (main human-AI reader experiment); 47 clinicians; 23 non-medical participants | Clinical photography | Multi-class classification of skin diseases, malignancy prediction | Human vs.  AI vs.  Human + AI | Diagnostic performance of AI-assisted skin disease classification | Process and experiential | Directly observed | AI assistance improved physician diagnostic accuracy and sensitivity for malignancy detection, highest performance observed in human–AI collaboration |
| Tschandl et al., 2020 (30) | Retrospective multi-reader human–AI interaction study | Experimental | 1,412 dermoscopic images, 302 clinician raters | Dermoscopy | Classification  + Triage | Human vs.  AI vs.  Human + AI | Diagnostic performance of human, AI, and human-AI collaboration for melanoma classification | Process and experiential | Directly observed | CNN assistance improved dermatologists' diagnostic accuracy, greatest benefit among less experienced dermatologists. |
| Lee et al., 2020 (31) | Retrospective reader study | Experimental | 1072 dermoscopic images of acral melanocytic lesions, 60 physicians | Dermoscopy | Classification | Human vs.  Human + AI | Diagnostic accuracy of CNN-assisted detection of acral lentiginous melanoma | Process and experiential | Directly observed | AI-assisted decision support improved diagnostic accuracy and interobserver concordance for ALM detection. |
| Marchetti et al., 2020 (32) | Cross-sectional reader study | Experimental | 150 dermoscopic images; 17 physicians (8 dermatologists, 9 residents) | Dermoscopy | Classification | Human vs.  Human + AI | Diagnostic accuracy of AI-assisted melanoma classification | Process and experiential | Directly observed + Model-based | Algorithm substitution for low-confidence assessments was associated with modeled improvement in dermatologist diagnostic performance. |
| Cerminara et al., 2023 (33) | Prospective clinical study | Clinical (real-world implementation) | 143 high-risk patients (1690 melanocytic lesions) | Clinical photography   + Dermoscopy | Melanoma risk classification + Decision support | Human vs.  Human + AI | Diagnostic accuracy of 2D-CNN, 3D-CNN, dermatologist assessment, and augmented intelligence for melanoma detection | Process and experiential | Directly observed | 3D-CNN outperformed 2D-CNN and matched dermatologist sensitivity, but limited specificity currently restricts the clinical utility of augmented intelligence. |

**Supplementary Table S2:** Characteristics of included studies and reported value-oriented outcomes**.**

**Supplementary Table Reference List:**

1.	Bramer EM, Chia C, Rentroia-Pacheco B, Tokez S, Pijnenborg L, Damman J, Amir A, Kumar D, Hollestein LM, Mooyaart AL, et al. Enhancing Early Detection of Metastatic Cutaneous Squamous Cell Carcinoma (CSCC): Integrating AI With Histopathological Assessments. *Exp Dermatol* (2025) 34:e70135. doi: 10.1111/exd.70135

2.	Aung TN, Liu M, Su D, Shafi S, Boyaci C, Steen S, Tsiknakis N, Vidal JM, Maher N, Micevic G, et al. Pathologist-Read vs AI-Driven Assessment of Tumor-Infiltrating Lymphocytes in Melanoma. *JAMA Netw Open* (2025) 8:e2518906. doi: 10.1001/jamanetworkopen.2025.18906

3.	Jones OT, Calanzani N, Scott SE, Matin RN, Emery J, Walter FM. User and Developer Views on Using AI Technologies to Facilitate the Early Detection of Skin Cancers in Primary Care Settings: Qualitative Semistructured Interview Study. *JMIR Cancer* (2025) 11:e60653. doi: 10.2196/60653

4.	Bossard C, Salhi Y, Khammari A, Brousseau M, Le Corre Y, Salhi S, Quéreux G, Chetritt JJ. Risk score stratification of cutaneous melanoma patients based on whole slide images analysis by deep learning. *J Eur Acad Dermatol Venereol JEADV* (2025) 39:1500–1509. doi: 10.1111/jdv.20538

5.	Gaube S, Biebl I, Engelmann MKM, Kleine A-K, Lermer E. Comparing preferences for skin cancer screening: AI-enabled app vs dermatologist. *Soc Sci Med* (2024) 349:116871. doi: 10.1016/j.socscimed.2024.116871

6.	Goessinger EV, Niederfeilner J-C, Cerminara S, Maul J-T, Kostner L, Kunz M, Huber S, Koral E, Habermacher L, Sabato G, et al. Patient and dermatologists’ perspectives on augmented intelligence for melanoma screening: A prospective study. *J Eur Acad Dermatol Venereol JEADV* (2024) 38:2240–2249. doi: 10.1111/jdv.19905

7.	Chanda T, Hauser K, Hobelsberger S, Bucher T-C, Garcia CN, Wies C, Kittler H, Tschandl P, Navarrete-Dechent C, Podlipnik S, et al. Dermatologist-like explainable AI enhances trust and confidence in diagnosing melanoma. *Nat Commun* (2024) 15:524. doi: 10.1038/s41467-023-43095-4

8.	Chanda T, Haggenmueller S, Bucher T-C, Holland-Letz T, Kittler H, Tschandl P, Heppt MV, Berking C, Utikal JS, Schilling B, et al. Dermatologist-like explainable AI enhances melanoma diagnosis accuracy: eye-tracking study. *Nat Commun* (2025) 16:4739. doi: 10.1038/s41467-025-59532-5

9.	Winkler JK, Blum A, Kommoss K, Enk A, Toberer F, Rosenberger A, Haenssle HA. Assessment of Diagnostic Performance of Dermatologists Cooperating With a Convolutional Neural Network in a Prospective Clinical Study: Human With Machine. *JAMA Dermatol* (2023) 159:621–627. doi: 10.1001/jamadermatol.2023.0905

10.	Escalé-Besa A, Yélamos O, Vidal-Alaball J, Fuster-Casanovas A, Miró Catalina Q, Börve A, Ander-Egg Aguilar R, Fustà-Novell X, Cubiró X, Rafat ME, et al. Exploring the potential of artificial intelligence in improving skin lesion diagnosis in primary care. *Sci Rep* (2023) 13:4293. doi: 10.1038/s41598-023-31340-1

11.	Sangers TE, Wakkee M, Moolenburgh FJ, Nijsten T, Lugtenberg M. Towards successful implementation of artificial intelligence in skin cancer care: a qualitative study exploring the views of dermatologists and general practitioners. *Arch Dermatol Res* (2023) 315:1187–1195. doi: 10.1007/s00403-022-02492-3

12.	Gomez Rossi J, Rojas-Perilla N, Krois J, Schwendicke F. Cost-effectiveness of Artificial Intelligence as a Decision-Support System Applied to the Detection and Grading of Melanoma, Dental Caries, and Diabetic Retinopathy. *JAMA Netw Open* (2022) 5:e220269. doi: 10.1001/jamanetworkopen.2022.0269

13.	Shoham G, Berl A, Shir-Az O, Shabo S, Shalom A. Predicting Mohs surgery complexity by applying machine learning to patient demographics and tumor characteristics. *Exp Dermatol* (2022) 31:1029–1035. doi: 10.1111/exd.14550

14.	Lim K, Neal-Smith G, Mitchell C, Xerri J, Chuanromanee P. Perceptions of the use of artificial intelligence in the diagnosis of skin cancer: an outpatient survey. *Clin Exp Dermatol* (2022) 47:542–546. doi: 10.1111/ced.14969

15.	Nelson CA, Pérez-Chada LM, Creadore A, Li SJ, Lo K, Manjaly P, Pournamdari AB, Tkachenko E, Barbieri JS, Ko JM, et al. Patient Perspectives on the Use of Artificial Intelligence for Skin Cancer Screening: A Qualitative Study. *JAMA Dermatol* (2020) 156:501–512. doi: 10.1001/jamadermatol.2019.5014

16.	Tong ST, Sopory P. Does integral affect influence intentions to use artificial intelligence for skin cancer screening? A test of the affect heuristic. *Psychol Health* (2019) 34:828–849. doi: 10.1080/08870446.2019.1579330

17.	Mei L-H, Cao M-K, Li J, Ye X-G, Liu X-D, Yang G. Deep learning in assisting dermatologists in classifying basal cell carcinoma from seborrheic keratosis. *Front Oncol* (2025) 15: doi: 10.3389/fonc.2025.1507322

18.	Diaz-Ramón JL, Gardeazabal J, Izu RM, Garrote E, Rasero J, Apraiz A, Penas C, Seijo S, Lopez-Saratxaga C, De la Peña PM, et al. Melanoma Clinical Decision Support System: An Artificial Intelligence-Based Tool to Diagnose and Predict Disease Outcome in Early-Stage Melanoma Patients. *Cancers* (2023) 15:

19.	Thomas L, Hyde C, Mullarkey D, Greenhalgh J, Kalsi D, Ko J. Real-world post-deployment performance of a novel machine learning-based digital health technology for skin lesion assessment and suggestions for post-market surveillance. *Front Med* (2023) 10:1264846. doi: 10.3389/fmed.2023.1264846

20.	Gregoor AMS, Sangers TE, Eekhof JA, Howe S, Revelman J, Litjens RJ, Sarac M, Bindels PJ, Bonten T, Wehrens R, et al. Artificial intelligence in mobile health for skin cancer diagnostics at home (AIM HIGH): a pilot feasibility study. *eClinicalMedicine* (2023) 60: doi: 10.1016/j.eclinm.2023.102019

21.	Marchetti MA, Cowen EA, Kurtansky NR, Weber J, Dauscher M, DeFazio J, Deng L, Dusza SW, Haliasos H, Halpern AC, et al. Prospective validation of dermoscopy-based open-source artificial intelligence for melanoma diagnosis (PROVE-AI study). *Npj Digit Med* (2023) 6:127. doi: 10.1038/s41746-023-00872-1

22.	Vollmer AS, Winkler JK, Kommoss K, Blum A, Tschandl P, Kränke T, Hofmann-Wellenhof E, Hofmann-Wellenhof R, Stolz W, Enk A, et al. The art of diagnosing rare skin tumors: Can DL-CNNs enhance dermatologists’ diagnostic accuracy? *Eur J Cancer* (2025) 228:115751. doi: 10.1016/j.ejca.2025.115751

23.	Metta C, Beretta A, Guidotti R, Yin Y, Gallinari P, Rinzivillo S, Giannotti F. Advancing Dermatological Diagnostics: Interpretable AI for Enhanced Skin Lesion Classification. *Diagnostics* (2024) 14:753. doi: 10.3390/diagnostics14070753

24.	Marsden H, Kemos P, Venzi M, Noy M, Maheswaran S, Francis N, Hyde C, Mullarkey D, Kalsi D, Thomas L. Accuracy of an artificial intelligence as a medical device as part of a UK-based skin cancer teledermatology service. *Front Med* (2024) 11: doi: 10.3389/fmed.2024.1302363

25.	Felmingham C, Pan Y, Kok Y, Kelly J, Gin D, Nguyen J, Goh M, Chamberlain A, Oakley A, Tucker S, et al. Improving skin cancer management with ARTificial intelligence: A pre-post intervention trial of an artificial intelligence system used as a diagnostic aid for skin cancer management in a real-world specialist dermatology setting. *J Am Acad Dermatol* (2023) 88:1138–1142. doi: 10.1016/j.jaad.2022.10.038

26.	Han SS, Kim YJ, Moon IJ, Jung JM, Lee MY, Lee WJ, Won C-H, Lee MW, Kim SH, Navarrete-Dechent C, et al. Evaluation of Artificial Intelligence–Assisted Diagnosis of Skin Neoplasms: A Single-Center, Paralleled, Unmasked, Randomized Controlled Trial. *J Invest Dermatol* (2022) 142:2353-2362.e2. doi: 10.1016/j.jid.2022.02.003

27.	Jahn AS, Navarini AA, Cerminara SE, Kostner L, Huber S, Kunz M, Maul J-T, Dummer R, Sommer S, Neuner AD, et al. Over-Detection of Melanoma-Suspect Lesions by a CE-Certified Smartphone App: Performance in Comparison to Dermatologists, 2D and 3D Convolutional Neural Networks in a Prospective Data Set of 1204 Pigmented Skin Lesions Involving Patients’ Perception. *Cancers* (2022) 14: doi: 10.3390/cancers14153829

28.	Ba W, Wu H, Chen WW, Wang SH, Zhang ZY, Wei XJ, Wang WJ, Yang L, Zhou DM, Zhuang YX, et al. Convolutional neural network assistance significantly improves dermatologists’ diagnosis of cutaneous tumours using clinical images. *Eur J Cancer* (2022) 169:156–165. doi: 10.1016/j.ejca.2022.04.015

29.	Han SS, Park I, Eun Chang S, Lim W, Kim MS, Park GH, Chae JB, Huh CH, Na J-I. Augmented Intelligence Dermatology: Deep Neural Networks Empower Medical Professionals in Diagnosing Skin Cancer and Predicting Treatment Options for 134 Skin Disorders. *J Invest Dermatol* (2020) 140:1753–1761. doi: 10.1016/j.jid.2020.01.019

30.	Tschandl P, Rinner C, Apalla Z, Argenziano G, Codella N, Halpern A, Janda M, Lallas A, Longo C, Malvehy J, et al. Human–computer collaboration for skin cancer recognition. *Nat Med* (2020) 26:1229–1234. doi: 10.1038/s41591-020-0942-0

31.	Lee S, Chu YS, Yoo SK, Choi S, Choe SJ, Koh SB, Chung KY, Xing L, Oh B, Yang S. Augmented decision-making for acral lentiginous melanoma detection using deep convolutional neural networks. *J Eur Acad Dermatol Venereol* (2020) 34:1842–1850. doi: 10.1111/jdv.16185

32.	Marchetti MA, Liopyris K, Dusza SW, Codella NCF, Gutman DA, Helba B, Kalloo A, Halpern AC, International Skin Imaging Collaboration. Computer algorithms show potential for improving dermatologists’ accuracy to diagnose cutaneous melanoma: Results of the International Skin Imaging Collaboration 2017. *J Am Acad Dermatol* (2020) 82:622–627. doi: 10.1016/j.jaad.2019.07.016

33.	Cerminara SE, Cheng P, Kostner L, Huber S, Kunz M, Maul J-T, Böhm JS, Dettwiler CF, Geser A, Jakopović C, et al. Diagnostic performance of augmented intelligence with 2D and 3D total body photography and convolutional neural networks in a high-risk population for melanoma under real-world conditions: A new era of skin cancer screening? *Eur J Cancer* (2023) 190:112954. doi: 10.1016/j.ejca.2023.112954
